# Les anomalies du fond d’œil au cours de la phase chronique de la maladie de Vogt Koyanagi Harada

**DOI:** 10.11604/pamj.2014.17.318.4139

**Published:** 2014-04-25

**Authors:** Hakima Elouarradi, Rajae Daoudi

**Affiliations:** 1 Service d'Ophtalmologie A de l'Hôpital des Spécialités, Université Mohammed V Souissi, Centre Hospitalier Universitaire, Rabat, Maroc

**Keywords:** Fond d’œil, maladie de Vogt Koyanagi Harada, Fundus, Vogt Koyanagi Harada disease

## Image en medicine

La maladie de Vogt Koyanagi Harada, est une panuvéite granulomateuse bilatérale avec décollement séreux rétinien, associés à des manifestations neurologiques (méningite lymphocytaire), auditives (surdité de perception) et dermatologiques (vitiligo, canitie et poliose des cils). Elle représente une cause majeure d'uvéite en Afrique du Nord. L’évolution de l'atteinte oculaire est caractérisée par la survenue d'une phase chronique, convalescente ou récurrente. Les lésions cicatricielles du fond d'oeil à la phase chronique du VKH sont fréquentes et variables: Dépigmentation diffuse du fond d'oeil avec Aspect en coucher de soleil (ou Sunset glow fundus), les cicatrices nummulaires atrophiques, migration et condensation pigmentaire, et fibrose sous rétinienne’ Nous rapportons le cas d'un patient âgé de 40 ans suivi pour maladie de VKH avec une mauvaise compliance au traitement, avec un examen du fond d'oeil objectivant une dépigmentation diffuse du fond d'oeil avec des lésions nummulaires d'atrophie choriorétinienne (A), des migrations pigmentaires au niveau de la macula responsables d'une baisse de l'acuité visuelle (B). Ces différentes lésions cicatricielles pigmentées décrites au cours des phases chroniques du VKH sont d'intérêt capital dans la mesure ou elles pourraient surtout aider au diagnostic rétrospectif de la maladie.

**Figure 1:**
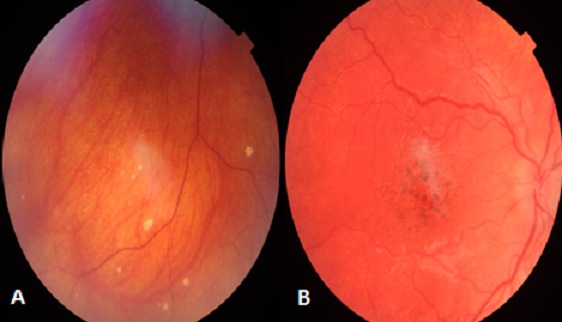
Aspect dépigmenté du fond d’œil:(A) lésions nummulaires d'atrophie choroiretinienne au niveau de la périphérie rétinienne (B) Remaniement pigmentaire au niveau de la macula

